# Baicalein Prevents 6-Hydroxydopamine-Induced Mitochondrial Dysfunction in SH-SY5Y Cells via Inhibition of Mitochondrial Oxidation and Up-Regulation of DJ-1 Protein Expression

**DOI:** 10.3390/molecules181214726

**Published:** 2013-11-27

**Authors:** Yue-Hua Wang, Hai-Tao Yu, Xiao-Ping Pu, Guan-Hua Du

**Affiliations:** 1 Department of Molecular and Cellular Pharmacology, School of Pharmaceutical Sciences, Peking University; Beijing 100191, China; 2 Beijing Key Laboratory of Drug Target Identification, Institute of Materia Medica, Chinese Academy of Medical Sciences & Peking Union Medical College, Beijing 100050, China; 3 State Key Laboratory of Bioactive Substance and Function of Natural Medicines, Institute of Materia Medica, Chinese Academy of Medical Sciences & Peking Union Medical College, Beijing 100050, China; 4 Jiangsu Kanon Pharmaceutical Co., Ltd, Lianyungang 222047, China

**Keywords:** baicalein, Parkinson’s disease, 6-hydroxydopamine, SH-SY5Y cells, brain mitochondria

## Abstract

Parkinson’s disease (PD) is a neurodegenerative disorder characterized by progressive loss of dopaminergic (DA) neurons at the substantia nigra. Mitochondrial dysfunction is involved in the mechanism of cell damage in Parkinson’s disease (PD). 6-Hydroxydopamine (6-OHDA) is a dopamine analog which specifically damages dopaminergic neurons. Baicalein has been previously reported to have potential in the treatment of PD. The purpose of the present study was to investigate the mechanism of action of baicalein against 6-OHDA injury in SH-SY5Y cells. The results showed that baicalein significantly alleviated alterations of mitochondrial redox activity and mitochondrial membrane potential induced by 6-OHDA in a dose-dependent manner in SH-SY5Y cells compared with vehicle group. Futhermore, baicalein decreased the production of ROS and upregulated the DJ-1 protein expression in SH-SY5Y cells. In addition, baicalein also inhibited ROS production and lipid peroxidation (IC_50_ = 6.32 ± 0.03 μM) in rat brain mitochondia. In summary, the underlying mechanisms of baicalein against 6-OHDA-induced mitochondrial dysfunction may involve inhibition of mitochondrial oxidation and upregulation of DJ-1 protein expression.

## 1. Introduction

Parkinson’s disease (PD) is a neurodegenerative disorder characterized by slowly progressive degeneration of dopamine (DA) neurons in the substantia nigra pars compacta, with subsequent damage of nerve terminals, accompanied by DA depletion in the striatum. Although the neuropathological hallmarks of PD are well described, the etiology remains still undefined. Notably, several observations suggest that mitochondrial dysfunction and oxidative stress are involved in the pathogenesis of PD [1,2,3].

Many lines of evidence suggest that mitochondrial dysfunction plays a central role in the pathogenesis of PD [4]. Mitochondrion is an essential organelle that supplies the cell with ATP through oxidative phosphorylation, synthesizes key molecules, and buffers calcium gradients [5]. It is not surprising that mitochondrial health is tightly regulated and associated with the homeostasis and aging of the organism [6]. Mitochondrial diseases often have an associated metabolic component, and consequently mitochondrial defects are expected in aging, and other energy-dependent disturbances [7]. In such disturbances, cellular oxidative damage caused by the generation of reactive oxygen species (ROS) that exceed the natural antioxidant activity is likely an initiating factor in aging [8]. ROS cause oxidative damage to proteins, lipids, and DNA and are one of the most prominent factors related to neurodegeneration. Therefore, regulation of redox signaling and inhibiting excess ROS generation would contribute greatly not only to extend longevity but also to ameliorate the progression of dopaminergic cell death seen in patients with PD [9].

The protein DJ-1 was first identified as a novel oncogene product [10] and was later found to be a causative gene product of a familial form of PD (PARK7) [11]. DJ-1 plays important roles in transcriptional regulation [12], anti-oxidative stress reaction [13,14,15,16], and the elimination of abnormal protein aggregates [17]. DJ-1 is a ubiquitous redox-responsive cytoprotective protein with diverse functions. In addition to its direct quenching ROS effect within neurons, DJ-1 may play a role of neuroprotection in a transcellular manner in chronic neurodegenerative diseases [18].

6-Hydroxydopamine (6-OHDA) is a dopamine analog, which specifically damages dopaminergic neurons either via uncoupling mitochondrial oxidative phosphorylation resulting in energy deprivation or alternatively, is associated with its ability to produce hydrogen peroxide, hydroxyl and superoxide radicals under physiological pH conditions [19]. Evidence demonstrates that 6-OHDA generates ROS and induces apoptosis in dopaminergic cells of rat substantia nigra [20]. It has also been reported that 6-OHDA inhibits complexes I and IV of the mitochondrial respiratory chain [21,22]. Therefore, 6-OHDA is used to investigate the cellular and molecular mechanisms underlying selective degeneration of dopaminergic neurons in PD. The SH-SY5Y cell line has become a popular cell model for PD research because this cell line possesses many characateristics of DAergic neurons [23]. It has been used as an *in vitro* model for the study of PD and to determine the effect of protective and therapeutic agents. It is thought that 6-OHDA induces toxicity that mimics the neuropathological and biochemical characteristics of PD in SH-SY5Y cells [24,25,26,27,28]. Therefore, the 6-OHDA-induced SH-SY5Y cell toxicity was used as a *vitro* PD model in our studies to investigate the possible protective effect of baicalein.

**Figure 1:**
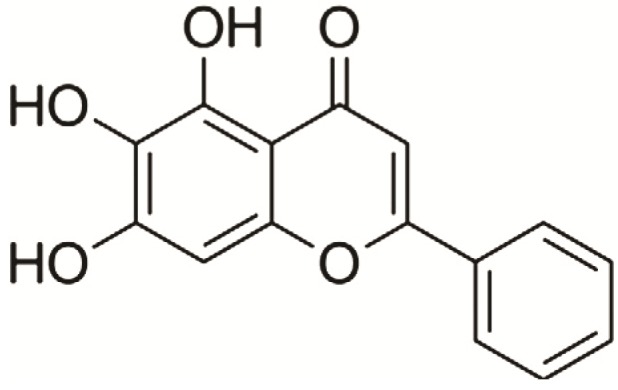
The chemical structure of baicalein.

Baicalein, a flavonoid obtained from the roots of the traditional Chinese herbal medicine Huangqin, *Scutellaria baicalensis* Georgi (Figure 1), has been widely used for treatment of inflammation, hypertension, cardiovascular disease, bacterial infection and cancer [29,30]. Our previous studies have shown that baicalein has anti-experimental Parkinsonism effects, especially against muscle tremors, in a mice model [31,32] and a rat model [33], however, the mechanisms and target protein(s) underlying this protective effect remain largely unknown. The purpose of this study was to explore the mechanism of action of baicalein against PD.

## 2. Results and Discussion

### 2.1. Effect of Baicalein on Morphology and Cell Viability in SH-SY5Y Cells Damaged by 6-OHDA

**Figure 2:**
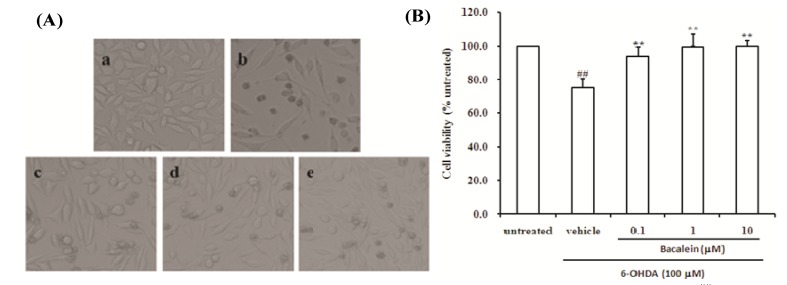
Effect of baicalein on the morphological changes and viability of SH-SY5Y cells induced by 6-OHDA. (**A**) Morphological changes were observed by light microscopy. Representative photographs showing control cells (a), vehicle cells (b), 6-OHDA + baicalein 0.1 µM (c), 6-OHDA + baicalein 1 µM (d), and 6-OHDA+baicalein 10 µM (e). (**B**) Cell viability was estimated by MTT assay.

It is known that 6-OHDA could selectively cause degeneration of the nigrostriatal dopaminergic neuronal pathway in several animals [22] and cells [34,35], so 6-OHDA-damaged SH-SY5Y cells were used as an *in vitro* PD model in our studies to investigate the possible mechanism of action of baicalein. As shown in Figure 2A, within 24 h of treatment with 6-OHDA alone, the majority of SH-SY5Y cells had undergone morphological changes such as membrane blebbing and cell shrinkage. Co-treatment with baicalein protected the cells from 6-OHDA damage.

Data were expressed as percent cell viability of untreated cells. Data are the mean ± SD, *n* = 4, ^##^ *p <* 0.01 *vs.* untreated group; ** *p <* 0.01 *vs.* vehicle group.

We also determined the cell viability by MTT assay, Treatment of SH-SY5Y cells with 6-OHDA alone resulted in an approximately 25% reduction in cell survival within 24 h, whereas co-treatment with 0.1, 1 and 10 µM baicalein all showed a reduction of 6-OHDA-mediated cytotoxicity (all *p* < 0.01. Figure 2B). These results indicate that the incubation of SH-SY5Y cells with baicalein effectively prevents 6-OHDA-induced cytotoxicity.

### 2.2. Baicalein Attenuates the Decrease of Mitochondria Redox Activity and the Collapse of Mitochondrial Membrane Potential Induced by 6-OHDA in SH-SY5Y Cells

**Figure 3:**
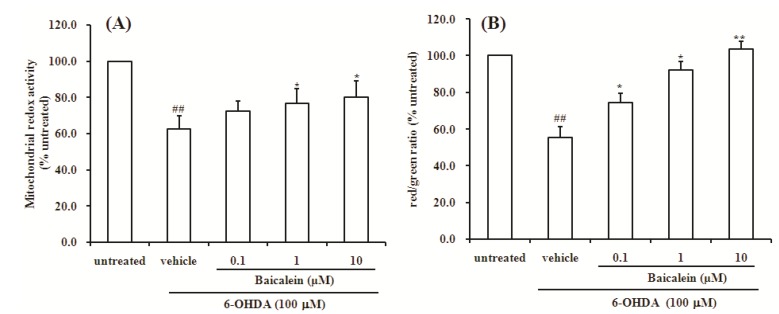
Effect of baicalein on mitochondrial redox activity and mitochondria membrane potential of SH-SY5Y cells induced by 6-OHDA. (**A**) Mitochondrial redox activity was assayed by resazurin; (**B**) Mitochondria membrane potential was assayed by JC-1 staining. Data were expressed as percentage of untreated cells.

Mitochondrial dysfunction has long been implicated in the pathogenesis of Parkinson’s disease (PD). The integrity of mitochondrial function is crucial for the maintenance of cell viability. Increasing evidence suggests that mitochondria are deeply involved in the production of reactive oxygen species through the electron carriers of the respiratory chain [36,37,38,39]. Mitochondrial dysfunction was detected as a decrease in mitochondrial redox activity and a loss in mitochondrial membrane potential (*Δψm).* Here, we used the resazurin staining method for the dectection mitochondrial redox activity and the JC-1 staining assay for the detection *Δψm* in SH-SY5Y cells. Rezazurin is a fluorescent indicator of mitochondrial function. JC-1 is sensitive to mitochondrial membrane potential, and the changes in the ratio between aggregate (red) and monomer (green) fluorescence can provide information regarding the mitochondrial membrane potential. Thus, resazurin and JC-1 are valuable analytical tools for examining mitochondrial function [40]. The results showed that 24 h of incubation with 6-OHDA significantly reduced mitochondria redox activity compared to the untreated group (*p* < 0.01, Figure 3A). On the other hand, co-treatment with 1 μM and 10 μM of baicalein significantly attenuated mitochondria redox activity impair induced by 6-OHDA (both *p* < 0.05, Figure 3A). As shown in Figure 3B, treatment with 100 µM of 6-OHDA for 24 h resulted in significant decrease of *Δψm* compared with untreated group (*p* < 0.01, Figure 3B).

Data are the mean ± SD, *n* = 4, ## *p <* 0.01 *vs.* untreated group; * *p <* 0.05, ** *p <* 0.01 *vs.* vehicle group.

However, the decrease of *Δψm* induced by 6-OHDA was significantly attenuated by co-treatment with baicalein in a dose-dependent manner. The present study indicates that mitochondrial redox activity decrease and mitochondrial membrane potential dissipation may play important roles in 6-OHDA induced dopaminergic neurotoxicity, and further provides that improvement of mitochondrial dysfunction may be a better way to slow progressive dopaminergic neurodegeneration commonly associated with PD.

### 2.3. Baicalein Reduces the Production of ROS Induced by 6-OHDA in SH-SY5Y Cells

**Figure 4:**
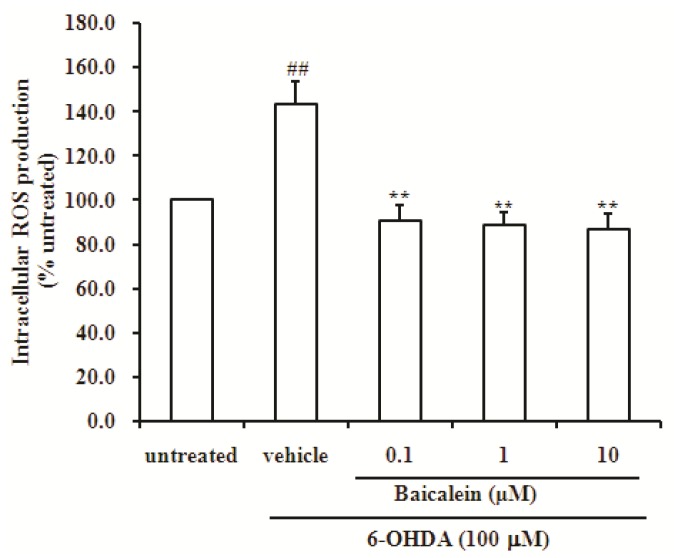
Effect of baicalein on generation of ROS induced by 6-OHDA in SH-SY5Y cells. Data were expressed as percentage of untreated cells.

6-OHDA is a highly reactive substance, which is readily auto-oxidized and oxidatively deaminated by monoamine oxidase, to give rise to ROS [41], which in turn, can cause DNA strand breaks, damage protein residues and initiate lipid peroxidation reactions [42]. The intracellular ROS level was determined by DCF fluorescence. SH-SY5Y cells treated with 6-OHDA showed a significant increase (about 1.4-fold) of intracellular ROS compared with untreated cells (*p <* 0.01, Figure 4). This increase was significantly attenuated by co-incubation with 0.1, 1 and 10 µM baicalein (all *p <* 0.01, Figure 4). This result indicates that the co-incubation of SH-SY5Y cells with baicalein effectively prevents 6-OHDA-induced the production of ROS (Figure 4). These results indicate that ROS plays an important role in the induction of neuronal damage by 6-OHDA, and baicalein could inhibit the production of ROS in SH-SY5Y cells, which indicates that the blockage of ROS by baicalein might be a very important factor for its neuroprotection.

Data are the mean ± SD, *n* = 4, ## *p* < 0.01 *vs*. untreated group; ** *p* < 0.01 *vs.* vehicle group.

### 2.4. Baicalein Up-Regulates DJ-1 Protein Expression in SH-SY5Y Cells

DJ-1 is known to play a key role in antioxidant and neuroprotection in neuronal cells [13,43,44]. DJ-1 regulates redox signaling kinase pathways and acts as a transcriptional regulator of antioxidative gene batteries. Therefore, DJ-1 is an important redox-reactive signaling intermediate controlling oxidative stress during age-related neurodegenerative processes. The anti-oxidant properties of DJ-1 lead to cytoprotection under oxidative stress conditions [45,46,47]. Thus, up-regulation of DJ-1 protein expression or augmenting DJ-1 activity might provide novel approaches to treating chronic neurodegenerative illnesses [18]. It is reported that DJ-1 binds to the mitochondrial complex I and plays a role in maintenance of mitochondrial complex I integrity [48].

**Figure 5:**
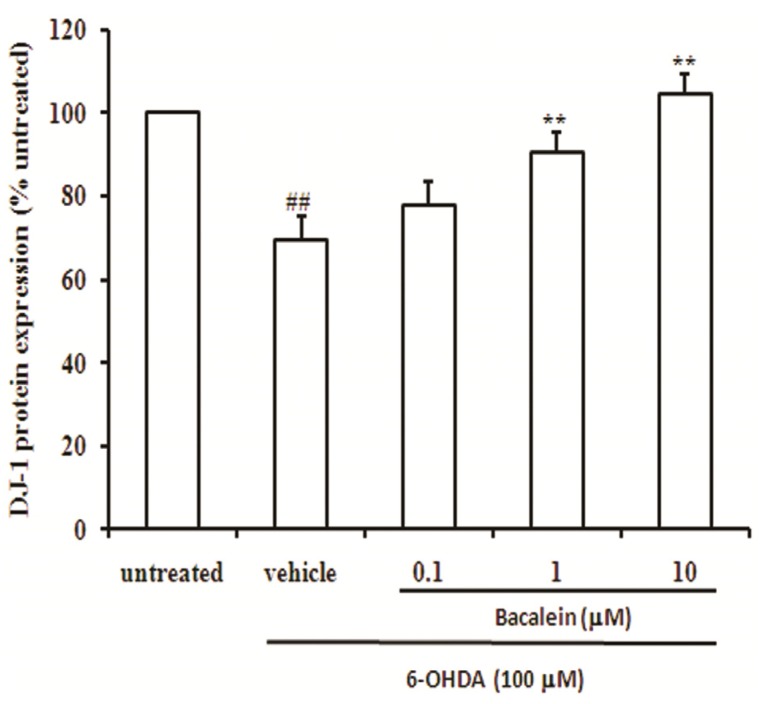
Effect of baicalein on DJ-1 protein expression in SH-SY5Y cells induced by 6-OHDA. Data were expressed as percentage of untreated cells.

As shown in Figure 5, treatment of SH-SY5Y cells with 6-OHDA alone for 24 h resulted in an approximately 22% reduction in DJ-1 protein (*p* < 0.01), whereas co-treatment with baicalein significantly increased DJ-1 protein expression in a dose-dependent manner in SH-SY5Y cells compared with vehicle group. This result suggests that baicalein effects reduction of cytotoxicity and mitochondrial dysfunction via up-regulation of DJ-1 protein. Our finding also indicates that DJ-1 is critical for mitochondrial function and may be the targeting protein for baicalein against PD.

Data are the mean ± SD, *n* = 4, ## *p* < 0.01 *vs.* untreated group; ** *p* < 0.01 *vs.* vehicle group.

### 2.5. Baicalein Suppresses the ROS Production Induced by 6-OHDA and Lipid Peroxidation in Rat Brain Mitochondria

A wide variety of ROS are produced in the course of the normal metabolism in biological systems and they have several important physiological functions, but their accumulation beyond the required amount can potentially damage lipids, proteins, and nucleic acids [49].

**Figure 6:**
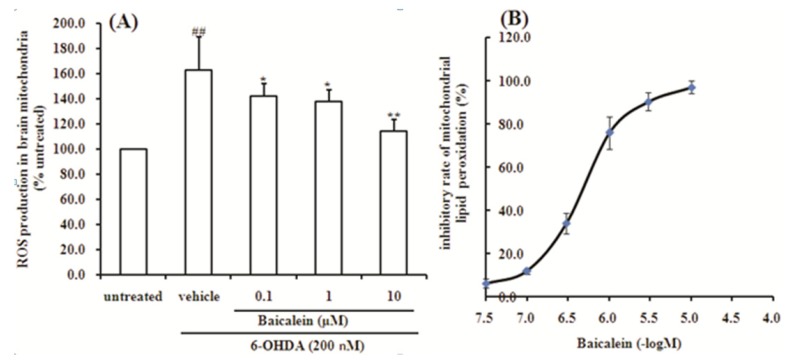
Effect of baicalein on generation of ROS induced by 6-OHDA and lipid peroxidation in rat brain mitochondria. (**A**) ROS production in rat brain mitochondria; (**B**) lipid peroxidation in rat brain mitochondria.

To observe the direct effect of baicalein on mitochondrial oxidative stress, reactive oxidant species level was measured in isolated rat brain mitochondria in further research. Baicalein exhibited significant protective effects on 6-OHDA-induced reactive oxidant species production, which were in accordance with the results obtained from the cellular study (Figure 6A). In addition, baicalein also inhibited the lipid peroxidation of brain mitochondria in a concentration-dependent manner (Figure 6B). The IC_50_ value is 6.32 ± 0.03 μM. Our results showed baicalein reduced the production of ROS in rat brain mitochondria and inhibit lipid peroxidation of rat brain mitochondria.

Data were expressed as percentage of untreated cells. Data are the mean ± SD, *n* = 4, ## *p* < 0.01 *vs.* untreated group; ** *p* < 0.01 *vs.* vehicle group.

## 3. Experimental

### 3.1. Materials

Bacalein was obtained from National Institutes for Food and Drug Control (Beijing, China). The SH-SY5Y cell line was obtained from Cell Culture Centre, Institute of Basic Medical Sciences, Chinese Academy of Medical Science (Beijing, China). DMEM and fetal bovine serum were purchased from Gibco (Grand Island, NY, USA). 6-OHDA, MTT, 2,7-dichlorodihydrofluorescein diacetate (H_2_DCF-DA) and resazurin were purchased from Sigma-Aldrich (St. Louis, MO, USA). JC-1 assay kit was purchased from Beyotime Institute of Biotechnology (Haimen City, Jiangsu, China). DJ-1 Cell-Based ELISA Kit was a product of the Abnova Company (Taipei City, Taiwan).

### 3.2. Cell Culture and Treatment

SH-SY5Y cells were grown in DMEM supplemented with 10% fetal bovine serum at 37 °C in a humidified atmosphere containing 5% CO_2_. Stock solution of 6-OHDA (100 mM, 1,000×) was dissolved in 0.2% ascorbic acid and aliquoted for storage at −20 °C. Before 6-OHDA was added to the culture medium, the stock solution was freshly diluted to the final concentration of 100 μM with serum-free DMEM medium. The cells were plated into 96-well plates at a density of 2 × 10^5^ cells/mL. After 70%–80% confluence, the cells were pre-incubated with different concentration of baicalein in a serum-free DMEM medium for 1 h. Then, 6-OHDA was added to the wells at a final concentration of 100 µM and incubated for another 24 h at 37 °C. In this study, the untreated group represented the control group which was treated with serum-free DMEM medium containing 0.0002% ascorbic acid, but without 6-OHDA. The vehicle group represented 6-OHDA-treated alone without baicalein.

### 3.3. Morphological Observations and Viability Assay

Morphological changes were observed under microscope. Cell viability was evaluated by the MTT assay [50]. Briefly, after treatment, MTT (0.5 mg/mL) was added to each well and incubated for 4 h at 37 °C. Then, the supernatant was removed and the formazan product obtained was dissolved in 100 µL dimethylsulfoxide (DMSO) with stirring for 15 min on a microtiter plate shaker and the absorbance was read at 540 nm using a Spectramax M5 microplate reader (Molecular Devices, Sunnyvale, CA, USA).

### 3.4. Measurement of Mitochondrial Redox Activity by Resazurin

Rezazurin is a fluorescent indicator of mitochondrial function. Upon oxidoreductase exposure in mitochondria, resazurin (blue and nonfluorescent) is reduced to resorufin (pink and highly florescent). The pink fluorescence intensity is examined at an excitation of 530 nm and an emission of 590 nm. The change rate in fluorescence intensity is associated with mitochondrial redox activity. After treatment, resazurin at final concentration of 5 µM were added into the wells and fluorescence intensity was examined at an excitation of 530 nm and an emission of 590 nm. Then, the plate was incubated for another 60 min then fluorescence was measured. The changing rate was represented as (F_60_ − F_0_)/F_0_ × 100%. F_60_, F_0_ referred to fluorescence of 60 min and 0 min [51].

### 3.5. Measurement of Mitochondrial Membrane Potential (Δψm) by JC-1

The fluorescent probe JC-1 (5,5',6,6'-tetrachloro-1,1',3,3'-tetraethylbenzimidazolylcarbocyanine iodide) was used to estimate of mitochondrial membrane potential (*Δψm*) [52]. After treatment, the culture medium was removed and loaded with JC-1 solution for 15 min at 37 °C in the dark. After two more rinses with Hank’s solution, fluorescence intensity of the red/green ratio was determined at an excitation of 490 nm and emission of 530 nm (green fluorescent monomers) and 590 nm (red fluorescent aggregates) respectively.

### 3.6. Determination of Intracellular ROS Level

Intracellular ROS level was measured using 2,7-dichlorofluorescein-dictate (H_2_DCF-DA) staining method [53]. After incubation with 6-OHDA, cells were loaded with 10 µM H_2_DCFHDA for 30 min at 37 °C in the dark. Cells were then analyzed on a Spectramax M5 microplate reader with excitation at 488 nm and emission at 525 nm.

### 3.7. DJ-1 Protein Expression Assay Using Cell-Based ELISA

After treatment, the cells were quenched, fixed and blocked. Primary antibodies specific for DJ-1 protein and GAPDH were added and allowed to bind to their respective epitopes. Then, HRP-conjugated secondary antibodies were added and incubated for 1.5 h at room temperature with gentle shaking. After washing, TMB solution was added to each well and incubated for 30 min at room temperature, and then the stop solution was added to each well and read OD at 450 nm immediately using the microplate reader. After finishing reading the absorbance at 450 nm, the plate was washed twice with Wash Buffer and twice with TBS and crystal violet was added to each well to bind with cell nuclei and give absorbance readings proportional to cell counts at 595 nm. The measured OD_450_ readings can be normalized using the OD_595 _values via the proportion (OD_450_/OD_595_).

### 3.8. Isolation of Rat Brain Mitochondria

Rat brain mitochondria were obtained by differential centrifugation [54]. Male Sprague-Dawley rats (250–300 g; Beijing Vital River Laboratory Animal Technology Co., Ltd; license: SCXK (JING) 2007-0001) were used in this study. All animal experiments were approved by the Laboratories Institutional Animal Care and Use Committee of Chinese Academy of Medical Sciences. Briefly, rats were decapitated and the brain was placed in beakers containing ice-cold isolation buffer (0.25 M sucrose containing 10 mM Tris–HCl, 1 mM EDTA, and 250 μg/mL BSA, pH 7.4). The brain tissue was weighed, repeatedly washed with the isolation buffer to remove adhering blood. Then, 10 volume (w/v) of homogenate was prepared in a glass homogenizer. The nuclei and cell debris were sedimented by centrifugation at 1,000 *g* for 10 min at 4 °C and discarded. The supernatant was collected for further centrifugation at 10,000 *g* for 10 min at 4 °C. The sediment were washed, gently suspended in the isolation medium and centrifuged at 10,000 *g* for 10 min at 4 °C for further purification of the mitochondria. Finally, the sedimented mitochondria were suspended in the above buffer at a concentration of 10–15 mg/mL. Mitochondrial protein concentration was determined by the Bradford method using BSA as a standard. Mitochondria were prepared fresh for each experiment and used within 4 h of isolation.

### 3.9. Detection of ROS Production in Rat Brain Mitochondria

ROS production in isolated rat brain mitochondria was monitored using the fluorescent probe H_2_DCF-DA. After incubation for 30 min with 100 μM 6-OHDA with or without baicalein pretreatment, mitochondria of different groups were incubated with 10 µM H_2_DCF-DA at 37 °C for another 30 min, and the fluorescence intensity was measured at an excitation wavelength of 488 nm and emission wavelength of 525 nm in a microplate reader.

### 3.10. Lipid Peroxidation of Rat Brain Mitochondria Induced by FeSO_4_-Cystine

Lipid peroxidation was determined by the formation of thiobarbituric acid reactive substances (TBARS) as described and modification. Briefly, rat brain mitochondria 100 µg/well in 0.2 M histidine buffer including FeSO_4_ 50 µM and cystine 500 µM was added into 96-well plate. Then, different concentration of baicalein were added and incubated at 37 °C for 30 min. The incubation was stopped by the addition of 1.0% thiobarbituric acid (TBA) solution and incubated for 30 min at 60 °C. The absorption was measured at 532 nm.

### 3.11. Statistical Analysis

The data were expressed as the means ± SD. Significance of differences between group means was determined by One-way analysis of variance (ANOVA) followed by *t*-test. A *p*-value < 0.05 was considered statistically significant.

## 4. Conclusions

Here we have demonstrated that baicalein had neuroprotective effects against 6-OHDA-induced cytotoxicity and mitochondrial dysfunction in SH-SY5Y cells and brain mitochondria. The results indicate that baicalein protected against 6-OHDA-induced mitochondrial dysfunction by reducing intracellular ROS and up-regulation of DJ-1 protein expression. However, further studies should be conducted on the detailed mechanisms of how baicalein could reduce ROS and up-regulate DJ-1 protein expression.
